# Targeting KasA: isosakuranetin derivatives as promising scaffolds for novel anti-tuberculosis agents against drug-resistant *Mycobacterium tuberculosis*

**DOI:** 10.3389/fbinf.2026.1777858

**Published:** 2026-03-31

**Authors:** B. Angitha, T. Amritha, Radul R. Dev, Rajesh Raju, C. V. Umesh, J. Abhithaj

**Affiliations:** 1 Centre for Integrative Omics Data Science, Yenepoya (Deemed to be University), Mangalore, Karnataka, India; 2 Department of Chemistry, MES Ponnani College, Ponnani, Kerala, India

**Keywords:** Chromolaena odorata, isosakuranetin, KasA, molecular dynamics, *Mycobacterium tuberculosis*

## Abstract

**Introduction:**

*Mycobacterium tuberculosis* remains a major global health threat due to the rising prevalence of multidrug-resistant (MDR) and extensively drug-resistant (XDR) strains, which limit the effectiveness of current therapies. The ß-keto-acyl carrier protein synthase (KasA), a key enzyme in the FAS-II pathway for mycolic acid biosynthesis, is a promising target for new anti-tuberculosis agents. *Chromolaena odorata*, a medicinal plant with reported antimicrobial and antituberculosis activity, is a rich source of bioactive flavonoids, including Isosakuranetin, which shows moderate anti-tuberculosis activity. Modifications in pharmacophores—such as functional groups, structural features, bond angles, and bond distances—can enhance the activity of these phytochemicals and improve their potential as drug leads.

**Methodology:**

A structure-based computational workflow was employed, including molecular docking, MM-GBSA binding energy calculations, ADMET evaluation, and 250 ns molecular dynamics simulations to investigate the binding affinity, stability, and pharmacokinetic profiles of Isosakuranetin and its derivatives against KasA.

**Results:**

The analysis revealed differential binding affinities and dynamic stabilities of Isosakuranetin derivatives. Isn_96 exhibited the strongest binding affinity (−7.921 kcal/mol), with favorable electrostatic and hydrophobic interactions involving residues HIS311, HIS345, and ASP273. Post-MDS MM-GBSA analysis confirmed its enhanced stability, displaying the highest binding free energy (−56.20 ± 6.90 kcal/mol). Pharmacokinetic predictions also indicated acceptable absorption and safety profiles.

**Discussion:**

These findings suggest that Isosakuranetin derivatives, particularly Isn_96, are promising scaffolds for the design of novel KasA inhibitors. Their strong binding affinity, dynamic stability, and favorable ADMET properties highlight potential efficacy against drug-resistant M. tuberculosis. The results emphasize the potential of plant-derived flavonoids as lead compounds and underscore the value of structure-based computational approaches in guiding anti-tuberculosis drug development.

## Introduction

Despite decades of global intervention, tuberculosis remains a major global health burden with over 10 million new cases and more than 1.5 million deaths annually (WHO, 2024). The emergence of Multi-Drug Resistant (MDR-TB) and Extensively Drug Resistant (XDR-TB) strains has significantly worsened the situation (Alibrahim et al., 2024)*. Mycobacterium tuberculosis* (Mtb) is undoubtedly the deadliest infectious agent in human history. It is responsible for the development of the chronic disease tuberculosis (TB), which is currently the most prevalent contagious agent-related cause of mortality worldwide (Inoyama et al., 2020). The primary root cause of transmission is the airborne spread of infectious aerosol droplets expelled by people with active pulmonary TB, enabling the pathogen to initiate infection upon inhalation. This pathogen’s success is critically dependent on its unique, highly impermeable lipid-rich cell wall, which is a robust defence against host immunity and most conventional antibiotics. The structural backbone of this barrier is mycolic acid, a long-chain C60-90 α-alkyl β-hydroxy fatty acid that confers pathogenicity, intrinsic antibiotic resistance, and persistence within human macrophages (Schiebel et al., 2013).

The Mycolic acid biosynthesis in *M. tuberculosis* is driven by two distinct but interconnected fatty acid synthesis pathways: the mammalian-like type I fatty acid synthesis (FAS-I) and bacterial type II (FAS-II) systems. FAS-I produces the short fatty acid precursor, which is elongated by the FAS-II system into long-chain meromycolates, the core component of mycolic acid (Swanson et al., 2009). Within the bacterial FAS-II machinery, the *β*-ketoacyl acyl carrier protein synthase (KasA) plays a pivotal role by catalyzing the chain elongation step required for mycolic acid formation. Importantly, KasA is unique to the bacterial FAS-II system and has no mammalian counterpart, making it an attractive selective target (Takayama et al., 2005). Since mycolic acids are essential for maintaining the structural integrity, pathogenicity, and intrinsic drug resistance of *M. tuberculosis*, inhibition of KasA effectively disrupts mycolic acid biosynthesis, leading to compromised cell wall formation and ultimately bacterial death (Maiolini et al., 2020).

KasA is the primary condensing enzyme in the FAS-II cycle, which catalyzes the Claisen condensation reaction that drives the progressive elongation of the meromycolate chain. Structurally, KasA functions as a homodimer with each monomer consisting of a core domain and a cap domain (Luckner et al., 2009) with a deep hydrophobic substrate-binding tunnel, which is crucial for accommodating the long acyl chains of meromycolate precursors. At the centre of this tunnel lies the conserved catalytic triad CYS171, HIS311 and HIS345, which facilitates the formation of a new C-C bond via acylation, decarboxylation and condensation mechanism (Rudraraju et al., 2022). During the catalytic process, the active site cysteine (CYS171) becomes acylated and is subsequently attacked by the decarboxylated malonyl-AcpM, leading to the formation of the *β*-ketoacyl-AcpM product that extends the fatty acyl chain by two carbon atoms. A C171Q KasA variation mimics the acylated enzyme intermediate by forming a hydrogen bond with the KasA oxyanion hole (KREMER et al., 2002). This acyl enzyme mimic is particularly valuable because several known KasA inhibitors, including Thiolactomycin (TLM) and platensimycin, exert their inhibitory activity by binding preferentially to the acyl-enzyme state of KasA enzyme. Incorporating these insights strengthens future efforts to design more potent and selective FAS-II inhibitors targeting KasA (Machutta et al., 2010).

The current anti-tubercular therapy recommended by the World Health Organization (WHO) primarily relies on first-line drugs, including Rifampicin, Isoniazid, Pyrazinamide, and Ethambutol (RHZE). These agents target distinct molecular pathways essential for *M. tuberculosis* survival. Isoniazid inhibits the enoyl-acyl carrier protein reductase (InhA) in the FAS-II system, thereby blocking the mycolic acid biosynthesis. Rifampicin inhibits RNA polymerase, suppressing transcription; Ethambutol interferes with arabinosyl transferase, preventing arabinogalactan layer formation in the cell wall, and pyrazinamide disrupts the membrane energetics and fatty acid synthesis under acidic intracellular conditions (Pai et al., 2016). Although these drugs form the cornerstone of TB treatment, their prolonged administration for six to 9 months often results in poor compliance and hepatotoxicity, contributing to the rise of multidrug-resistant (MDR) and extensively drug-resistant (XDR) strains. Second-line drugs such as Levofloxacin, Moxifloxacin, Amikacin, Cycloserine, Ethionamide and Para-aminosalicylic acid (PAS) are used in resistant cases, but they are less potent, more toxic and require even longer treatment durations. In recent years, new agents including Bedaquiline, Delamanid and clofazimine have expanded the therapeutic arsenal against drug-resistant TB (Monde et al., 2023). However, emerging resistance to these drugs and their limited accessibility underscore the urgent need for novel molecular targets and scaffolds capable of effectively circumventing existing resistance mechanisms.

Despite its importance, the molecular mechanism of KasA inhibition and its interaction with novel scaffolds remain partially characterized (Schiebel et al., 2013). Several FAS-II inhibitors have been explored to target this pathway, including KasA-specific inhibitors thiolactomycin (TLM), cerulenin and their synthetic analogues, which block the acyl elongation step by occupying the hydrophobic substrate tunnel of KasA. Previous work with natural compounds like thiolactomycin (TLM, the co-crystallized ligand in 4C70) established that small molecules can effectively block the hydrophobic substrate tunnel, thereby validating it as a druggable hotspot within the FAS-II system.

The phytochemical filtering of different parts of *Chromolaena odorata* revealed the presence of alkaloids, cyanogenic glycosides, flavonoids, saponins and tannins. In recent years, flavonoids in particular have gained considerable interest due to their ability to disrupt the Mtb cell wall and exhibit broad antimicrobial, anti-inflammatory, anti-allergic and anti-cancer properties (Pawar et al., 2020). The chemical composition and antimicrobial potential of *C. odorata* extracts have been widely documented, and among the four major flavanones present in flowers, Isosakuranetin has been reported to possess notable antimycobacterial activity against *M. tuberculosis* (Suksamrarn et al., 2004). These attributes make Isosakuranetin, along with its derivatives, a promising scaffold for structure-based optimization targeting the FAS-II system. However, Isosakuranetin derivatives have not been extensively studied against KasA, leaving a significant gap in understanding their potential as selective FAS-II inhibitors (Vo et al., 2025).

Fragment-based modifications of Isosakuranetin allow the introduction of diverse alkyl, aryl, and functional groups to enhance hydrophobic interactions, hydrogen bonding capacity, and overall binding affinity, all of which are critical parameters in rational drug design. However, despite the availability of natural and synthetic KasA inhibitors, the precise manner in which the KasA active site accommodates bulky mycolic acid precursors and newly engineered scaffolds remains poorly understood. This knowledge gap limits the development of more potent and selective inhibitors.

Therefore, the present study applies an integrated *in silico* workflow combining molecular docking, binding affinity prediction, ADMET profiling, and molecular dynamics simulations to screen a library of Isosakuranetin derivatives. This approach aims to identify a lead compound capable of effectively occupying and stabilizing within the KasA catalytic tunnel, thereby providing mechanistic insights crucial for the rational design of next-generation antitubercular agents.

## Methodology

### Protein preparation

The three-dimensional structure of the KasA homodimer from *M. tuberculosis* was retrieved from the RCSB-PDB using the accession code PDB:4C70. This crystal structure represents a CYS171 Gln (C171Q) mutant in complex with TLM4. The protein was prepared and processed using the Protein Preparation Wizard in the Maestro (version 14.6) software. Initially, preprocessing corrected the structural defects and added missing atoms or side chains essential for accurate simulation. The structure was then optimized using the OPLS4 force field to assign appropriate protonation states and partial charges. All crystallographic water molecules were deleted to prevent interference with ligand binding during grid generation. Finally, the protein was minimized to obtain a refined, stable conformation with the lowest potential energy.

### Ligand preparation

The three-dimensional structure of Isosakuranetin was retrieved from the PubChem database, and its derivatives were synthesized using Library synthesizer, a Java-based *Insilico* tool. Compound Library information and sketching are an essential part of drug lead identification or drug lead optimization. Compound Library Synthesis through this software is a multi-step procedure. For convenience, the first loaded fragment may be called the primary fragment. Each fragment can evolve a lot of molecules. In this work, Isosakuranetin were used as a primary fragment for generating three different libraries. The first library evolved from Isosakuranetin. Similarly, second and third libraries were prepared from designed derivatives of Isosakuranetin. Systematic modifications were introduced through the substitution or addition of functional groups and aromatic ring systems at specific positions on the parent structure. These structural variations were designed in accordance with relevant physicochemical and drug-likeness parameters, leading to the generation of an expanded and chemically diverse molecular library. This approach enabled the creation of a substantial number of structurally related candidate molecules for subsequent evaluation. The generated library was refined based on their Log P values, the number of hydrogen bond donors (H.B.D.), and hydrogen bond acceptors (H.B.A.) using this applet.

The Ligprep tool in the Maestro version 14.6 software (Schrodinger Molecular Modelling Suite) was used for the preparation of ligand sets. Each ligand was generated with one stereochemical isoform per structure, and protonation state was assigned at pH 7.0 using the Epik ionizer. The optimized low-energy 3D structures were produced as tautomerized, desalted forms using the OPLS4 force field (Johnston et al., 2023).

### Molecular Docking

The ligands were screened using the GLIDE module of the Schrodinger Molecular Modelling Suite, which employs a virtual screening method based on grid-based ligand docking (Friesner et al., 2004). The active site residues of the prepared KasA homodimer were used to define the receptor grid for the protein structure, with dimensions of 20 × 20 × 20 Å centred on the centroid of the co-crystallized ligand (Manthattil et al., 2026). The molecular docking was validated by redocking the co-crystallized ligand TLM4 into the defined grid. The Extra Precision (XP) of the Glide module was employed to redock the prepared ligands into the active site of the KasA. The Root Mean Square Deviation (RMSD) was calculated, and additional validation analyses were performed to confirm the ligand’s orientation within the binding site. The virtual screening workflow was executed on the generated grid using the prepared ligands to examine their binding affinity on the KasA active site. According to Friesner et al. (2006), the virtual screening method started with High Throughput Virtual Screening (HTVS), followed by Standard Precision (SP), Extra Precision (XP) and finally prime-MM GBSA (Jacobson et al., 2004). Following each refinement, the batch of ligands was narrowed down to the best ones based on binding energy and the highest docking score.

### ADMET

To guarantee the toxicity profile, effectiveness, and safety of the repurposed drug candidates, their pharmacokinetic and pharmacodynamic characteristics were assessed via ADMET analysis using ADMETlab 3.0 (https://admetlab3.scbdd.com). This web-based tool predicts key parameters related to Absorption, Distribution, Metabolism, Excretion, and Toxicity. The SMILES notation of each compound was extracted and then used as input for the ADMET predictions.

### Molecular Dynamics Simulation (MDS)

Using Schrödinger’s Desmond module, the selected protein-ligand complex underwent a 250 ns molecular dynamics simulation to evaluate the binding affinity and residence time of the ligand at the active site (Bowers et al., 2006). The protein was first prepared by solvating it in an orthorhombic box filled with TIP3P water molecules using the System Builder. The structure was minimized to optimize the protein-ligand complex. Sodium and chloride ions were added to neutralize the system. The simulation ran under a constant number of particles, pressure, and temperature (NPT) conditions, applying the Nose-Hoover chain thermostat and the Martyna-Tobias-Klein barostat to maintain temperature and pressure stability. These settings enabled an accurate molecular dynamics simulation of the highest-affinity compound. Trajectory analyses included Root Mean Square Deviation (RMSD), Root Mean Square Fluctuation (RMSF), ligand interaction profiling, radius of gyration (Rg), Solvent Accessible Surface Area (SASA), and post-simulation MM-GBSA binding free energy calculations of the protein-ligand complex.

### POST MDS-MMGBSA

The Schrodinger suite’s Prime Module was used to compute the free energy of ligand binding to protein for the trajectories using the Python script thermal_mmgbsa.py. The MMGBSA calculation was performed for a 2500-frame trajectory for all three complexes and for the standard inhibitor. The binding free energy was calculated by extracting frames at a time interval of 250 ns. A force field of OPLS4 was employed for the process, and a step size of 100 was used to build the input structures for the Prime module.

## Results

### Molecular docking

**FIGURE 1:**
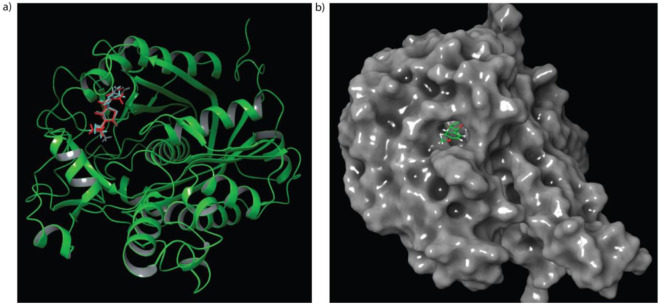
**(a)** The ligand TLM4 in crystal (Cyan) and redocked into the active site (Red) **(b)** The surface view of KasA with Isosakuranetin.

The TLM4 (C171Q) mutant of KasA was used as the receptor structure in this study, as this mutation reliably mimics the acyl-enzyme intermediate, thereby stabilizing the key interactions within the Oxyanion hole. This physiologically relevant conformation enables more accurate docking validation and ligand-binding assessment. The docking score, representing the predicted binding affinity, was used to rank the compounds, where a more negative value indicates a stronger binding affinity. Binding energy (MM-GBSA) represents the estimated free energy change upon protein-ligand complex formation, with a more negative value indicating a more thermodynamically stable and favourable interaction. To ensure the validity of the docking workflow, the native ligand was redocked into the TLM4 (C171Q) active site (Figure 1). The redocked pose closely overlapped with the crystallographic conformation, yielding an RMSD of 0.59 Å and interactions with HIS311 and HIS345 residues. Following redocking validation, the workflow was applied to evaluate the binding affinity of Isosakuranetin and its six derivatives against the *β*-ketoacyl-ACP synthase (KasA) of *M. tuberculosis.* Molecular docking was performed, and both docking score and MM-GBSA, along with detailed ligand contacts on key KasA residues, were considered together to ensure a thorough and reliable assessment of inhibitory potential.

The docked compounds exhibited scores ranging from −7.921 to −5.712 kcal/mol. The standard inhibitor displayed a docking score of −6.665 kcal/mol with a binding energy of −50.375 kcal/mol. Isosakuranetin exhibited a docking score of −6.973 kcal/mol, corresponding to a binding energy of −37.6162 kcal/mol, indicating a moderate affinity to the target.

Among the derivatives, Isn_184_A67 exhibited a docking score of −7.304 kcal/mol and binding energy of −18.0557 kcal/mol, forming a salt bridge with ASP273, *π*-*π* stacking with PHE 404 and two hydrogen bonds with ASN408 and HIE311. Isn_184_A137 had a docking score of −7.183 kcal/mol and binding energy of −21.6845 kcal/mol, interacting with five residues (ASP273, ASN 408, GLU322, PHE404, HIE311), while Isn_184 displayed a docking score of −6.554 kcal/mol and binding energy of −21.0532 kcal/mol forming three hydrogen bonds (GLY403, HIE311, HIE 345) and a *π*-*π* interaction with PHE404 as shown in Table 1. Isn_172 exhibited a docking score of −5.984 kcal/mol and binding energy −46.4347 kcal/mol, forming hydrogen bonds with GLY318, HIE311, and HIE345 and *π*-*π* stacking with HIE311. The final derivative had a docking score of −5.712 kcal/mol and binding energy of −41.9293 kcal/mol, with a single hydrogen bond involving HIE345.

**TABLE 1:** Docking score, MMGBSA dg bind and interacting residues of Isosakuranetin and its derivatives.

Derivatives	Docking score (kcal/mol)	MMGBSA dg bind (kcal/mol)	Interacting residues
Standard inhibitor	−6.665	−50.3751	HIE 311, HIE 345
Isosakuranetin	−6.973	−37.6162	GLY 403, THR 313
Isn_96	−7.921	−36.2836	ASN 408, ASP 273, PHE 404, GLN 171
Isn_184_A67	−7.304	−18.0557	ASN 408, ASP 273, PHE 404, HIE 311
Isn_184_A137	−7.183	−21.6845	ASN 408, ASP 273, GLU 322, PHE 404, HIE 311
Isn_184	−6.554	−21.0532	GLY 403, PHE 404 HIE 311, HIE 345
Isn_172	−5.984	−46.4347	GLY 318, HIE 311, HIE 345
Isn_184_A148_08	−5.712	−41.9293	HIE 345

**FIGURE 2:**
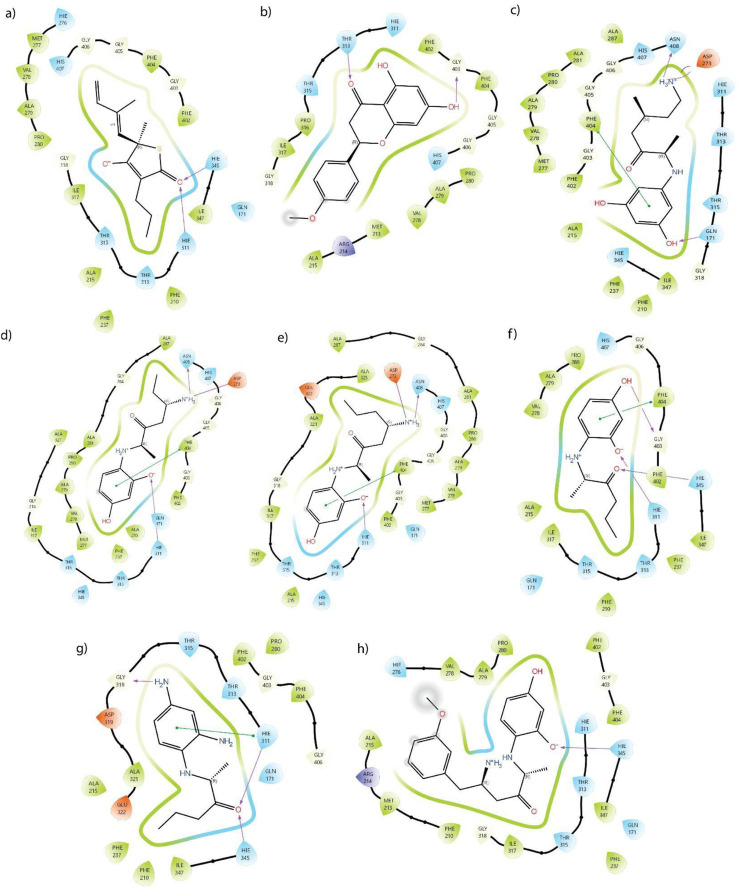
Ligand Interaction of **(a)** Standard inhibitor, **(b)** Isosakuranetin, **(c)** Isn_96, **(d)** Isn_184_A67, **(e)** Isn_184_A137, **(f)** Isn_184, **(g)** Isn_172, **(h)** Isn_184_A148_08.

Interestingly, Isn_96 outperformed other derivatives with the highest favourable binding affinity with a docking score of −7.921 kcal/mol and binding energy of −36.2836 kcal/mol. It formed multiple non-covalent interactions, including *π*-*π* stacking and hydrogen bonds with PHE 404, ASN408, ASP273, and HIE311 residues at the active site and inhibitory region, suggesting a robust potential to inhibit KasA. Based on the superior docking score and favourable binding energy, compounds were subjected to ADMET analysis to predict their pharmacokinetic properties. The interaction patterns of all ligands with KasA are illustrated in Figure 2.

### ADMET

The ADMET analysis was performed on Isosakuranetin, its derivatives and on the standard inhibitor. This evaluation was conducted before the Molecular Dynamics simulation to ensure that candidates possessed favourable pharmacokinetic and pharmacodynamic properties. Furthermore, Isosakuranetin is predicted to have a high rate of hepatic metabolism, which leads to rapid drug clearance from the bloodstream in addition to predicted hematotoxicity. In contrast, its derivatives exhibited more favourable and diverse ADMET characteristics, as summarised in Table 2.

**TABLE 2:** The ADMET profile of Isosakuranetin derivatives and reference standard inhibitor.

ADMET profiles	Standard inhibitor	Isn_96	Isn_184_A67	Isn_184_A137	Isosakuranetin	Isn_184	Isn_172	Isn_184_A148_08
Molecular weight (g/mol)	238.1	266.16	252.15	266.16	286.08	223.12	221.15	344.17
PAINS	0	0	0	0	0	0	0	0
nHA	2.0	5.0	5.0	5.0	5.0	4.0	4.0	6.0
nHD	1.0	5.0	5.0	5.0	2.0	3.0	5.0	5.0
nRot	4.0	7.0	6.0	7.0	2.0	5.0	5.0	8.0
TPSA	37.3	95.58	95.58	95.58	75.99	69.56	81.14	104.81
logS	−2.936	−1.716	−1.593	−2.835	−4.158	−2.977	−2.132	−3.396

Lipinski rule	Accepted	Accepted	Accepted	Accepted	Accepted	Accepted	Accepted	Accepted
Caco-2 permeability (log (cm/s))	−4.521	−5.744	−5.923	−5.964	−4.968	−4.977	−4.942	−5.926
Pgp-inhibitor	+++	—	—	—	+++	—	—	—
Pgp-substrate	—	+++	+++	+++	—	-	++	+++
HIA (HIA ≥ 30%)	—	—	—	—	—	—	—	—
PPB	98.2%	34.4%	26.4%	29.7%	96.9%	72.9%	61.6%	48.6%
VDss (L/kg)	1.743	2.192	2.917	3.238	0.987	0.734	1.408	2.441
BBB	—	—	—	—	—	—	–	—
CLplasma (mL/min/kg)	7.706	9.976	5.163	5.168	7.945	5.456	7.588	5.076
T1/2 (hours)	0.406	1.345	1.59	1.495	1.004	1.486	0.91	1.532
hERG blockers	0.018	0.097	0.058	0.09	0.194	0.025	0.03	0.142
Hematotoxicity	0.309	0.155	0.033	0.029	0.208	0.102	0.695	0.021
Carcinogenicity	0.445	0.536	0.191	0.086	0.568	0.488	0.777	0.169
DILI	0.86	0.201	0.307	0.283	0.84	0.325	0.712	0.266
Human hepatotoxicity	0.513	0.538	0.727	0.876	0.748	0.396	0.318	0.911
Nephrotoxicity	0.426	0.313	0.636	0.764	0.683	0.268	0.614	0.799

The prediction probability values: 0–0.1 (---), 0.1–0.3 (--), 0.3–0.5 (−), 0.5–0.7 (+), 0.7–0.9 (++), and 0.9–1.0 (+++).

Among the derivatives, Isn_96 displayed the most balanced ADMET profile, showing relatively high aqueous solubility (−1.716), low plasma protein binding (34.4%), moderate half-life and low predicted toxicity, indicating good bioavailability and safety. When it comes to Isn_184_A67, it has the most favourable pharmacokinetic properties with low overall toxicity risk, longest half-life (1.59 h), lowest PPB of 26.4% and low plasma clearance (5.163). Its primary problems are poor CaCO2 permeability and strong Pgp-substrate status. Then, following the other derivatives, Isn_184_A137 shares the advantages of low PPB (29.7%) and a long half-life of 1.495 h. It also demonstrates the lowest predicted carcinogenicity (0.086) and hematotoxicity (0.029) scores among the entire derivatives. However, it shows an increase in the hepatotoxicity score (0.876), which raises the safety concern.

The compound Isn_184 additionally exhibited favourable ADMET characteristics with the lowest toxicity scores and a favourable half-life (1.486 h) with weak Pgp-substrate (−), though it has moderate PPB (72.9%).

Conversely, the remaining derivatives and the standard inhibitor exhibited significant liabilities. Despite having the lowest plasma clearance (5.076) and a long half-life (1.532 h), it has the highest predicted human hepatotoxicity score (0.911) and a high P-gp substrate status. Due to its markedly high hematotoxicity (0.695) and carcinogenicity (0.777), Isn_172 is not considered for further development. The standard inhibitor exhibited moderate solubility (−2.936), high plasma protein binding (98.2%) and moderately high hepatic metabolism, resulting in a short half-life (0.406 h). It also showed moderate hepatotoxicity (0.513) and drug-induced liver injury risk, indicating limited systemic exposure and potential liver safety concerns. Despite these limitations, it served as a control compound for evaluating repurposed derivatives.

Thus, based on the overall pharmacokinetic profile and favourable docking score, Isn_96, Isn_184_A67, Isosakuranetin, and the standard inhibitor were selected for further molecular dynamics simulation to evaluate the stability and binding behaviour of the complex.

### Molecular dynamic simulation

#### Root mean square deviation (RMSD)

During MD simulations, the Root Mean Square Deviation (RMSD) of a ligand-protein complex is widely used to evaluate the structural stability of the protein backbone (C*α*) by measuring the average displacement of individual atoms over time (Henna et al., 2025). The average C*α* RMSD and standard deviation of apo KasA were around (3.27 ± 1.34 Å). As summarised in Table 3, the ligand-bound systems exhibited C*α* RMSD values of 2.65 ± 0.45 Å for the Isn_96 complex, 4.75 ± 0.85 Å for the standard inhibitor, 3.49 ± 1.69 Å for Isn_187_A67, and 3.62 ± 0.48 Å for Isosakuranetin.

**TABLE 3:** Average and standard deviation of protein-ligand complex from RMSD and RMSF.

Compounds	PL-RMSD	RMSF
Isn_96	2.65 ± 0.45	0.91 ± 0.83
Standard inhibitor	4.75 ± 0.85	1.06 ± 1.01
Isn_184_A67	3.49 ± 1.69	1.52 ± 1.81
Isosakuranetin	3.62 ± 0.48	1.14 ± 1.21
Protein only	3.27 ± 1.34	1.49 ± 1.74

**FIGURE 3:**
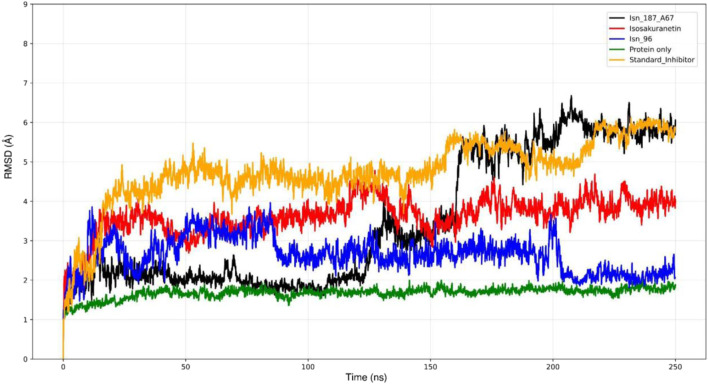
Combined C*α* RMSD graph of Isn_96-KasA complex, standard inhibitor-KasA complex, Isn_184_A67-KasA complex, protein_only, Isosakuranetin-KasA complex.

Throughout the 250 ns simulation, the RMSD trajectory of the KasA backbone (green line) remained relatively stable (Figure 3). Among the four ligand-bound complexes, Isn_96 exhibited the lowest average RMSD (2.65 Å) along with the smallest standard deviation, suggesting a highly stable and consistently maintained binding pose. In contrast, the standard inhibitor showed the highest C*α* RMSD (4.75 ± 0.85 Å), indicating the least stable interaction with KasA. Isn_184_A67 produced an intermediate mean C*α* RMSD but with the largest standard deviation (1.69 Å), reflecting a highly dynamic and inconsistent binding orientation. Isosakuranetin displayed a moderate deviation (3.62 Å) with a relatively small and stable standard deviation of 0.48 Å.

Overall, the C*α* RMSD analysis clearly indicates that Isn_96 provides the greatest structural stability to the KasA protein, as supported by its lowest RMSD values and minimal fluctuations across the simulation period.

#### Root Mean Square Fluctuation (RMSF)

The Root Mean Square Fluctuation (RMSF) provides insight into the flexibility and dynamic stability of individual amino acid residues within the protein-ligand complex during molecular dynamics (MD) simulations (Dcunha et al., 2026). It reflects the average deviation of a residue from its mean position over the simulation trajectory. A lower RMSF value corresponds to more rigid and stable regions, whereas a higher RMSF value indicates greater flexibility or local motion. The average RMSF values for each protein-ligand complex are summarised in Table 3.

**FIGURE 4:**
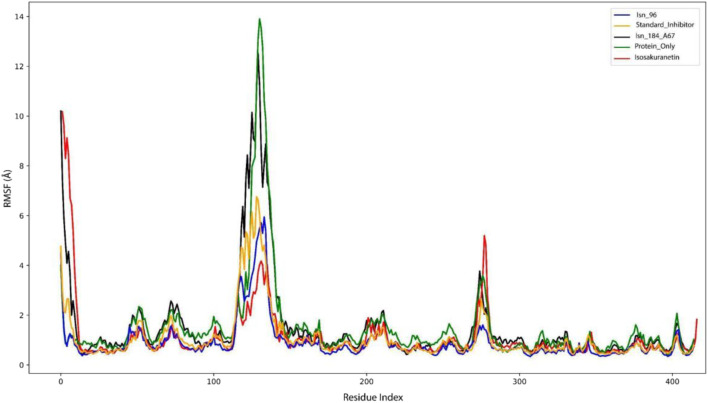
Combined RMSF graph of Isn_96, standard Inhibitor, Isn_184_A67, protein_only, Isosakuranetin-KasA complex.

The C*α* RMSF of the Isn_96 profile reveals the notable fluctuations in specific residue regions (Figure 4). Prominent peaks are observed between the residues 110–150 (≈3.8 Å) and 270–280 (≈3.2 Å), indicating enhanced flexibility in these regions. These peaks correspond to regions of strong protein-ligand interaction and are typically associated with loop regions, unstructured tails or active site residues, which are inherently more flexible. In contrast, the residues within 150–250 and 300–400 ranges exhibit low fluctuations, signifying structural stability and association with core secondary elements such as *α*-helices and *β*-sheets.

Similarly, considering the RMSF of the standard inhibitor, there was a fluctuation ranging from 4.0 Å to 4.5 Å throughout the simulation. The most notable peaks were seen at the residues 40–60 (≈2.0 Å),110–150 (≈4.5 Å), 260–280 (≈2.8 Å), indicating regions with more flexibility. The fluctuations are mainly caused by the conformational changes that occur during the ligand interaction at the loop and the binding site. A minor rise was also observed near the 400 residues (≈1.8 Å), suggesting limited flexibility at the terminal region. Conversely, the area between 150–350 and 300–380 exhibited a low C*α* RMSF value, indicating that they belong to stable secondary elements such as *α*-helix and *β*-sheets. This overall pattern indicates that despite the localized flexibility in certain loop regions, the protein backbone remains largely stable throughout the simulation.

The average RMSF of Isn_184_A67 was found to be approximately 1.52 ± 1.81 Å, indicating an overall moderately stable C*α* backbone throughout the simulation. RMSF values across the trajectory ranged from 0.6 Å to 6.4 Å. Most of the residues exhibited fluctuations below 2.0 Å, reflecting overall structural rigidity and stable folding during the simulation period. Notably, few pronounced peaks were observed at the N-terminus and within the region spanning residues 120–130, where the RMSF value reached up to 6.4 Å. These regions likely correspond to loop or coil segments, which typically display greater flexibility compared to the α helical and *β*-sheet domains. Additionally, slight fluctuations were detected around residues 250–310 along with a minor rise near residue 400, whereas the mid-section (residues 150–250) remained comparatively stable with RMSF values generally below 1.6 Å, indicating the structural integrity and rigidity of the secondary structure throughout the simulation.

Finally, the C*α* RMSF profile of the parent compound over the 250 ns MD demonstrates that the majority of residues exhibit limited fluctuation below 1.5 Å. However, two distinct loop regions displayed elevated flexibility. The first major peak occurs around residues 120–140, where the RMSF value reaches approximately 4.0 Å. A second prominent peak was observed within the residue, ranging from 270 to 295, with fluctuations rising to nearly 5.0 Å. This region shows a rigid ligand-binding interaction for stabilization within the binding pocket. In contrast, residues around 200–400 exhibit lower RMSF values reflecting the stable and rigid structure of the protein. Overall, the elevated fluctuations at the interaction site indicate conformational changes are essential for maintaining proper binding and functional dynamics.

#### Solvent Accessible Surface Area (SASA)

**FIGURE 5:**
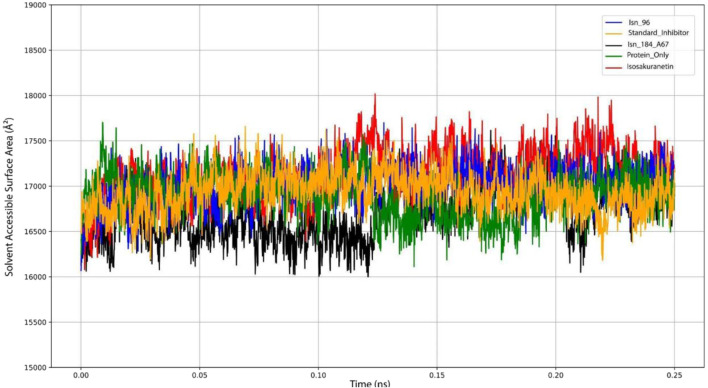
Graph plotting the Solvent Accessible Surface Area (SASA) of Isn_96, Standard inhibitor, Isn_187_A67, Protein only, Isosakuranetin.

Solvent Accessible Surface Area (SASA) analysis was performed to evaluate the degree of solvent exposure and structural compactness of KasA in the presence of different ligands. SASA quantifies the portion of the protein surface accessible to solvent molecules (John et al., 2025). Lower SASA values indicate a more compact structure, whereas larger values reflect greater solvent exposure. The SASA fluctuations throughout the simulation are illustrated in Figure 5.

Each ligand-bound compound displayed distinct SASA patterns. The KasA–Isn_96 complex showed an average SASA of 17,009.51 Å^2^, slightly higher than the standard inhibitor (16,901.52 Å^2^), suggesting a marginal increase in solvent exposure upon binding. The Isosakuranetin complex displayed the highest SASA value (17,143.08 Å^2^), indicating a more expanded structural state with increased interaction with solvent molecules. In contrast, Isn_184_A67 showed a notably lower SASA value (16,712.86 Å^2^), reflecting enhanced structural compactness and more efficient packing of the protein throughout the simulation. Additionally, the protein-only system exhibited an average SASA of 17,208.43 Å^2^, serving as a baseline reference for comparing ligand-induced structural changes.

#### Radius of gyration (Rg)

**FIGURE 6:**
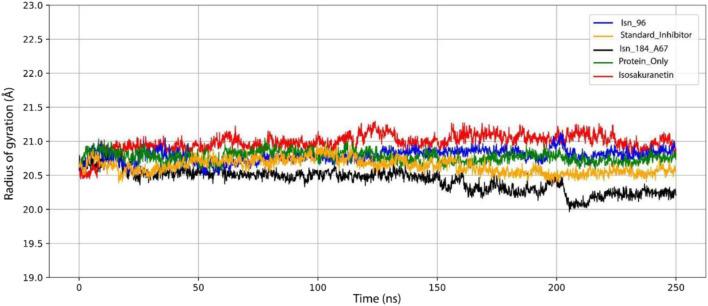
Graph plotting the Radius of Gyration (Rg) of Isn_96, Standard inhibitor, Isn_184_A67, Isosakuranetin, Protein only.

The radius of gyration (Rg) is an essential parameter for assessing the mobility and stiffness of a protein. The standard inhibitor, KasA, Isn_96, Isosakuranetin, Protein only and Isn_184_A67 systems exhibited average Rg values of 20.63 Å, 20.77 Å, 20.79 Å, 20.98 Å, 20.93 Å and 20.42 Å, respectively (Figure 6). The Rg value reflects the structural compactness of the protein, with a higher Rg indicating reduced compaction, and a lower Rg denotes greater compactness. Among all the compounds, Isn_184_A67 displayed the lowest average Rg value (20.42 Å), indicating the highest degree of structural compactness and suggesting that this ligand stabilizes the KasA conformation most effectively during the simulation. In contrast, Isn_96 showed a moderately higher Rg value (20.79 Å), reflecting a slightly more expanded structural state compared to Isn_184_A67. Although Isn_96 does not induce the greatest level of compactness, its Rg profile still lies within a stable range, indicating that the KasA–Isn_96 complex maintains overall structural integrity throughout the MD trajectory.

#### Protein-ligand contact

**FIGURE 7:**
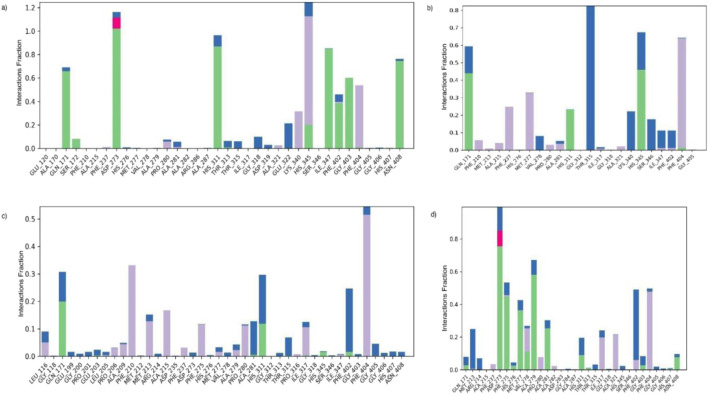
The protein-ligand contact of **(a)** Isn_96, **(b)** Standard inhibitor, **(c)** Isosakuranetin, and **(d)** Isn_184_A67.

The protein ligand contact plot illustrates the interaction fraction between the ligand and key protein residues throughout the molecular dynamics (MD) simulation trajectory (Thaikkad et al., 2025). Such analysis is essential for elucidating the stability and key intermolecular contacts within the protein-ligand complex. In the KasA-Isn_96 complex (Figure 7A), the catalytic tunnel residues HIS311 and HIS345 maintained strong interactions (including hydrogen bonds and hydrophobic interactions) with Isn_96 for approximately 86% and 92% of the simulation time, respectively. During the 250 ns MD Simulation, KasA-Isn_96 formed stable hydrogen bonds predominantly with GLN171, ASP 273 and ASN408, which persisted for 34%, 72% and 74% of the trajectory, respectively.

The interaction graph (Figure 7B) depicts the residue contact frequency for standard inhibitor ligand complexes across the trajectory. Among these interactions, THR315 exhibited the highest fraction of interaction through water bridges, persisting for about 80% of the simulation period. Likewise, PHE404 exhibited sustained contact with the ligand for about 68% of the simulation time. Residues such as GLN171 and HIS345 displayed a mixed-mode binding pattern reflecting their simultaneous involvement in polar and non-polar contacts that contribute to the overall stabilization of the complex.

For Isn_184_A67 (Figure 7D), the profile demonstrates an interaction fraction close to 100% for PHE273, mainly dominated by hydrogen bonding (∼75%), supported by moderate hydrophobic and minor water-bridge interactions. The ligand additionally forms hydrogen bonds with residues such as PHE275 (∼44%) and ALA279 (∼48%), along with notable hydrophobic and water-mediated contacts at PHE402 and PHE404, further enhancing the stability of the Isn_184_A67-KasA complex.

Analysis of the parent compound, Isosakuranetin (Figure 7C), reveals that the stability of the complex is primarily maintained by a strong hydrophobic interaction. Among these, PHE404 shows the highest contribution with an interaction fraction of 100%. Similarly, PHE210 also contributes significantly, with an interaction fraction of 33%. In addition to hydrophobic contacts, the ligand forms polar and hydrogen-bonding interactions with residues such as GLN171 and HIS311. This residue provides persistent polar stabilization during the simulation. The ligand also exhibits a strong polar contact with PHE402. Overall, the combination of extensive hydrophobic interactions and selected polar contacts establishes a robust interaction network that supports the structural stability of the protein–ligand complex throughout the simulation.

In summary, the MD interaction profile indicates that Isn_96 forms strong and persistent contacts with key catalytic residues of KasA, particularly HIS311, HIS345, ASP273, GLN171, and ASN408. These interactions occupy and stabilize the active site tunnel, thereby restricting substrate access and hindering its enzymatic activity, reinforcing its role as the most promising inhibitory candidate among the compounds.

### Post MDS MMGBSA

The binding free energy of the complex was calculated using the MM-GBSA method, which provides insights into the stability and affinity of the protein-ligand interaction. The binding free energy values for KasA in complex with the standard inhibitor, Isn_96, Isn_184_A67, and Isosakuranetin, as presented in Table 4 along with their contributing factors, were determined to be −38.68 ± 8.07 kcal/mol, −56.20 ± 6.90 kcal/mol, −49.67 ± 8.84 kcal/mol, and −36.54 ± 7.79 kcal/mol, respectively, along with their contributing energy components.

**TABLE 4:** POST MDS MM-GBSA of the top hit compounds.

Compound	Standard inhibitor	Isn_96	Isn_184_A67	Isosakuranetin
Energy contributing factor				
r_psp_MMGBSA_dG_Bind	−38.68 ± 8.07	−56.20 ± 6.90	−49.67 ± 8.84	−36.54 ± 7.79
r_psp_MMGBSA_dG_Bind_Coulomb	−8.81 ± 15.61	−96.98 ± 16.85	−74.65 ± 18.78	−8.18 ± 5.75
r_psp_MMGBSA_dG_Bind_Covalent	1.14 ± 0.64	2.55 ± 1.36	1.68 ± 0.96	0.78 ± 0.75
r_psp_MMGBSA_dG_Bind_Hbond	0.77 ± 0.35	−3.50 ± 0.54	−1.70 ± 0.33	−0.39 ± 0.48
r_psp_MMGBSA_dG_Bind_Lipo	−11.61 ± 2.06	−8.27 ± 1.77	−14.07 ± 2.42	−10.46 ± 1.84
r_psp_MMGBSA_dG_Bind_Solv_GB	18.13 ± 12.94	87.81 ± 12.90	74.34 ± 17.08	13.71 ± 3.07
r_psp_MMGBSA_dG_Bind_vdW	−36.77 ± 3.78	−36.21 ± 2.61	−34.66 ± 3.79	−30.16 ± 4.76

Among these, Isn_96 exhibited the most negative ΔG value (−56.20 kcal/mol), indicating the strongest and most favourable interaction with the KasA binding site. It also showed the highest Coulomb interaction contribution (−96.98 ± 16.85 kcal/mol), which is a major factor supporting its potency and selectivity. The superior affinity of Isn_96 arises mainly due to its highly favourable Coulombic contribution (−96.98 ± 16.85 kcal/mol), the strongest among all compounds, indicating a strong electrostatic interaction with key active-site residues.

Isn_184_A67 also showed a good binding energy (−49.67 kcal/mol), supported by both electrostatic and hydrophobic interactions. The Van der Waals interaction was consistent across all complexes, with Isn_96, Isn_184_A67 and the standard ligand showing comparable values. In contrast, the desolvation energy was unfavourable for all the complexes with moderate hydrogen bond contribution.

Thus, the result clearly highlights Isn_96 as the most promising candidate exhibiting a balance of binding energy, strong electrostatic forces, favourable Van der Waals interaction and stabilized hydrogen bonding.

## Discussion

Over the past 10 years, tuberculosis has remained one of the most widespread and persistent infectious diseases affecting millions of individuals annually and posing a major public health challenge. The disease is caused by *Mycobacterium tuberculosis,* a pathogen equipped with several virulence and pathogenicity factors, including a highly impermeable lipid-rich cell wall and strong intracellular survival mechanisms that contribute to its antimicrobial resistance. The rise of multiple drug-resistant strains has reduced the effectiveness of conventional therapies, emphasizing the need for effective antimicrobial agents as essential components in the treatment of TB (Veeramuthu et al., 2024).

Currently, the standard treatment for tuberculosis involves a combination of first-line antimicrobial drugs such as isoniazid, rifampicin, ethambutol and pyrazinamide, administered over a prolonged treatment course of at least 6 months (Bakare et al., 2022). Although these drugs have been effective for decades, their clinical success is often limited by several drawbacks. Long treatment duration leads to poor patient compliance, increasing the likelihood of treatment failure and resistance development. Moreover, many of these drugs are associated with serious adverse effects, including hepatotoxicity, peripheral neuropathy, gastrointestinal disturbances and hypersensitivity reactions, which further compromise patient adherence. The growing emergence of multidrug-resistant tuberculosis (MDR-TB) and extensively drug-resistant tuberculosis (XDR-TB) strains has further reduced the overall effectiveness of existing therapeutic regimens, underscoring the urgent need for new drug targets and innovative therapeutic strategies (Gandhi et al., 2010).

Natural compounds have been explored as a therapeutic option against tuberculosis due to their broad antimicrobial activity, structural diversity and lower likelihood of inducing severe side effects compared to conventional drugs. Many phytochemicals, microbial metabolites and marine-derived compounds possess strong inhibitory effects against *M. tuberculosis* by targeting essential biological processes (Maioli et al., 2020). Their ability to modulate multiple pathways makes them a promising candidate against multidrug-resistant strains. One such compound, the flavanone Isosakuranetin and its derivatives, has attracted research interest due to its notable properties and its potential to inhibit the key molecular targets of *M. tuberculosis*, positioning it as a strong lead candidate for drug development.

In this study, an *in silico* drug repurposing approach was employed to investigate the therapeutic potential of Isosakuranetin and its derivative against *M. tuberculosis.* Computational techniques, including Molecular docking, ADMET prediction, MMGBSA and Molecular dynamics simulation, were used to predict the binding affinity and stability of these compounds against the essential KasA enzyme involved in mycolic acid synthesis. Docking results revealed that several derivatives displayed stronger binding affinity than the parent compound and reference ligand inhibitor. ADMET prediction further demonstrated that most derivatives possessed favourable pharmacokinetic characteristics supporting their suitability for oral administration. During the sectional evolution procedure, a distinct effort was taken to introduce hydrophobic and hydrophilic groups to fragments. Hydrophilic groups such as the hydroxyl (-OH) group and the amino group -NH2 have a significant role in the interaction between drug molecules and receptor protein. In this study, OH groups produced more interaction than -NH2 groups. Among these, the synthesis of Isn_96 from Isosakuranetin is synthetically feasible through a rational multistep transformation strategy, beginning with demethylation of the methoxy (–OCH_3_) substituent to generate the corresponding phenolic hydroxyl group, enabling further functionalization. This is followed by oxidative ring opening of the flavanone C-ring to produce a substituted acetophenone intermediate, providing a versatile platform for side-chain introduction. The ketone functionality is subsequently converted to an α-haloketone and subjected to nucleophilic substitution with protected 3-aminopropylamine to install the amino linker. Subsequent chain extension and functionalization may be achieved through Michael addition or reductive amination reactions. Alternatively, a convergent synthetic route may be employed, wherein the side chain is synthesized separately and subsequently coupled with the catechol intermediate to obtain the target compound. Isn_96 also stands out with superior bioavailability, reduced toxicity and more drug-like properties. Molecular dynamics simulation performed on selected derivatives, along with the parent compound and the standard inhibitor, revealed that Isn_96 formed the most stable interaction with KasA. It exhibited low RMSD fluctuations, restricted residue flexibility around the catalytic tunnel and favourable binding energy values of −56.20 kcal/mol, indicating strong inhibitory potential. These combined findings support Isosakuranetin derivatives, particularly Isn_96, as a promising lead molecule for the development of new anti-tuberculosis agents.

However, as this study is solely based on computational analysis, the molecule shows good synthetic accessibility, indicating that progression to experimental validation is feasible. Therefore, the next phase of the study should involve chemical synthesis followed by comprehensive *in vitro* and *in vivo* evaluation to confirm biological efficacy and safety. Compared to currently available antimycobacterial drugs, which typically require prolonged treatment duration, Isn_96 exhibited lower predicted toxicity and has a better effect than the existing drugs. However, this hypothesis requires further validation through detailed *in vitro* assays, pharmacokinetic profiling, and controlled *in vivo* studies to substantiate its antimycobacterial activity and safety profile.

## Conclusion

The current study employed a structure-based computational framework to identify and evaluate potential inhibitors of the KasA enzyme, a vital component of the FAS-II pathway in *M. tuberculosis.* Through sequential molecular docking, MM-GBSA calculation, ADMET prediction and extensive Molecular Dynamic Simulation (250 ns), Isn_96 was identified as the most promising inhibitory scaffold. The compound demonstrated robust binding affinity (−7.921 kcal/mol), consistent interaction with catalytic residues (HIS 311, HIS 345, and ASP 273) and favourable pharmacokinetic properties. This performance was further supported by its highest binding free energy (−56.20 kcal/mol), establishing Isn_96 as a potent inhibitor of KasA. Overall, Isn_96 represents a promising lead for inhibiting KasA in tuberculosis. However, these computational findings require further validation through *in vitro* assays and *in vivo* studies to confirm the therapeutic potential and biological efficacy of the identified KasA inhibitors.

## Data Availability

The original contributions presented in the study are included in the article/supplementary material, further inquiries can be directed to the corresponding authors.
